# *Astragalus mongholicus* Bunge and Panax Notoginseng Formula (A&P) Combined With Bifidobacterium Contribute a Renoprotective Effect in Chronic Kidney Disease Through Inhibiting Macrophage Inflammatory Response in Kidney and Intestine

**DOI:** 10.3389/fphys.2020.583668

**Published:** 2020-11-27

**Authors:** Tan Rui-Zhi, Diao Hui, Li Jian-Chun, Zhong Xia, Wang Xiao-Jia, Wen Dan, Fan Jun-Ming, Wang Li

**Affiliations:** ^1^Research Center for Integrated Chinese and Western Medicine, Affiliated Traditional Medicine Hospital, Southwest Medical University, Luzhou, China; ^2^Department of Nephrology, Affiliated Hospital of Southwest Medical University, Luzhou, China; ^3^Department of Nephrology, Affiliated Hospital of Chengdu Medical College, Chengdu, China

**Keywords:** *Astragalus mongholicus* Bunge and Panax notoginseng formula, bifidobacterium, macrophage, CKD, Mincle

## Abstract

There is increasing evidence that Chronic Kidney Disease (CKD) can cause intestinal dysfunction, which in turn aggravates the progression of kidney disease. Studies have shown that the immune response of macrophage plays an important role in promoting inflammation in kidney and intestine of CKD. *Astragalus mongholicus* Bunge and Panax notoginseng formula (A&P) is a widely used traditional medicine for the treatment of CKD in China, however, the underlying mechanism is largely unclear. In this study, we aimed to explore the role of A&P and Bifidobacterium combination treatment in regulation of inflammatory response of macrophage in kidney and intestine of CKD mouse, as well as the potential molecular mechanism. We established a CKD mouse model with 5/6 nephrectomy and a macrophage inflammatory cellular model with LPS and urotoxin *in vivo* and *in vitro*. The results showed that A&P combined with Bifidobacterium significantly reduced the expression and secretion of IL-1β, IL-6, TNFα, and MCP-1 in kidney and blood, as well as in inflammatory macrophage. Interestingly, A&P combined with Bifidobacterium strongly improved the intestinal flora and protected the intestinal barrier. Notably, the maintainer of macrophage polarization, Mincle, was activated in kidney and intestine of CKD mouse as well as in urotoxin stimulated macrophage, that was effectively inhibited by the treatment of A&P and Bifidobacterium combination. Overexpression of Mincle by genetic modification can abolish the inhibitory effects of A&P combined with Bifidobacterium on inflammation in urotoxin stimulated RAW264.7 cells. In summary, these findings demonstrated that A&P combined with Bifidobacterium can protect kidney against CKD by down-regulating macrophage inflammatory response in kidney and intestine via suppressing Mincle signaling, which provides a new insight in the treatment of CKD with traditional medicine.

## Introduction

Chronic Kidney Disease (CKD), which is defined as the presence of renal structural and/or functional abnormalities for at least 3 months, has been widely regarded as an important public health problem. Recent epidemiological surveys revealed high levels of prevalent incidence of CKD worldwide which varies from 5 to 13% among countries (De Nicola and Minutolo, 2016). Considering the great medical and social burden, it casts urgent importance to the prevention, diagnosis and treatment of this disease.

In the advanced stage of chronic kidney disease, metabolic waste cannot be excreted by the intestine and kidney, and is accumulated in the body in large amounts, which may further enter the intestinal wall through blood vessels. Meanwhile, increased urotoxin in blood after kidney injury also enter the intestine wall, which promotes fundamental changes of the living environment of intestinal epithelium and intestinal flora, leading to an increase of the number of pathogenic bacteria and a reduction of beneficial bacteria, which directly or indirectly promotes the entry of toxins into intestinal epithelial cells, causing severe damage to the structure and function of intestinal barrier. Therefore, the permeability of the intestinal epithelium is greatly increased, which in turn causes the intestinal toxins to invade into the blood and accelerates the progression of CKD (Vaziri et al., 2013; Vanholder and Glorieux, 2015; Sommer et al., 2017; Hugenholtz and de Vos, 2018). The intestinal mucosal immune system is one of the largest immunological compartments in human body, and any destruction of the microbiome may cause an imbalance of the body’s immune system. Therefore, using modern medical methods to explore the mechanism of the intestinal microecological balance and the interaction between intestinal mucosal barrier function and CKD through animal model *in vivo* and cellular model *in vitro* are new directions worth exploring (Crespo-Salgado et al., 2016; Nallu et al., 2017).

Studies have shown that when an organ is injured, it starts an immune response and activates a variety of immune cells, including neutrophils, dendritic cells (DC), macrophages, natural killer cells (NK), T lymphocytes and regulatory T cells. Among them, macrophages are important innate immune cells with phagocytosis (Alikhan and Ricardo, 2013). Polarization of macrophages is divided according to their function (Murray and Wynn, 2011). Macrophages that secrete pro-inflammatory factors and exert pro-inflammatory functions are called M1 macrophages. On the contrary, macrophages that play a role in reducing inflammation and promoting tissue repair are called M2 macrophages (Barros et al., 2013; Peng et al., 2017; Soldano et al., 2018). Transition of the macrophage polarization is widespread in the occurrence and development of inflammatory diseases such as kidney disease, intestinal disease, obesity, and some cardiovascular disease (Bain and Mowat, 2014; De Schepper et al., 2018), in which the imbalance of macrophage polarization can reflect the inflammatory state of microenvironment of local tissues. Therefore, we believe that understanding the mechanism underlying polarization imbalance in intestinal macrophage is helpful for the treatment of CKD.

Mincle, macrophage-induced c-type lectin (Clec4e), is a transmembrane pattern recognition receptor involved in innate immunity and mainly expressed in innate immune cells, such as macrophages, dendritic cells, B cells and neutral Granulocytes (Patin et al., 2017; Lim and Lappas, 2019). Related studies have shown that Mincle is strictly regulated by TLR4/NF-κB signaling. Mincle plays a role in maintaining the M1 phenotype of pro-inflammatory macrophage in acute kidney disease (Hui et al., 2020). In the cisplatin-induced mouse AKI model, inhibition of Mincle on macrophage reduced the activity of M1 type macrophage in kidney, thereby improving kidney damage in mice (Inoue, 2017; Li et al., 2017; Wahi et al., 2019). Our previous research reported that isoliquiritigenin inhibited unilateral ureteral obstruction (UUO)-induced kidney damage by down-regulating the inflammation and fibrosis maintained by Mincle (Liao et al., 2020). However, the role of Mincle in chronic kidney disease and whether Mincle can be used as a therapeutic target for CKD are not clearly studied.

*Astragalus mongholicus* Bunge and Panax notoginseng formula is prescribed under the guidance of traditional Chinese medicine theory. It consists of Panax notoginseng, *Astragalus mongholicus* Bunge, Angelica sinensis, *Achyranthes bidentata* Blume, and *Ecklonia kurome* Okamura. Clinical studies have found that the combination of A&P and basic treatment can significantly improve the renal function and quality of life of patients with diabetic nephropathy and CKD (Hui et al., 2020; Wen et al., 2020). *Astragalus mongholicus* Bunge and Panax notoginseng are the main ingredients in this formula, which have high medicinal value. *Astragalus mongholicus* Bunge can improve metabolism, turnover of serum and liver proteins, immunity, cardiac function, as well as has the effects of lowering blood sugar, anti-tumor, anti-fatigue and antibacterial (Fu et al., 2014). Panax notoginseng is a widely used Chinese medicine, which can affect the central nervous system, circulatory system, digestive system, urinary system, reproductive system and immune system. It also has anti-aging, anti-tumor and anti-inflammatory effects (Wang et al., 2016). Previous study has found that in acute kidney injury, A&P can improve renal inflammation in mice by inhibiting the Mincle/NF-κB signaling pathway and M1 macrophage activity (Hui et al., 2020). Therefore, we try to reveal the effects and potential mechanism of A&P on CKD.

The probiotic Bifidobacterium is clinically used for diarrhea, constipation, indigestion and bloating caused by intestinal flora disorders, as well as adjuvant treatment of endotoxemia. Patients with intestinal dysfunction can take the preparation of Bifidobacteria to directly supplement the normal physiological bacteria of the human intestine, thereby adjusting the balance of intestinal microecology and rebuilding the integrity of intestinal barrier (Koppe et al., 2015; Jia et al., 2018; Tsai et al., 2019). From the point of immunology, bifidobacterium can improve the immune function of intestine (Cigarran Guldris et al., 2017), and promote the function of digestion and absorption of intestine. However, the use of Bifidobacteria alone in the treatment of CKD is controversial, whether the combination of Bifidobacteria and the traditional Chinese medicine formula A&P can enhance the improvement of chronic kidney disease remains to be studied.

In this present study, we used a novel 5/6 nephrectomy induced CKD mouse model *in vivo* and an inflammatory macrophage model *in vitro* to study the protective effects of A&P on kidney and intestine of CKD mice and explore whether A&P inhibited inflammation of kidney and intestine in CKD through regulating Mincle signaling, that may provides a new option for the treatment of CKD.

## Materials and Methods

### *Astragalus mongholicus* Bunge and Panax Notoginseng Formula and Bifidobacterium

*Astragalus mongholicus* Bunge and Panax notoginseng formula is a traditional Chinese medicine formula used clinically to treat kidney diseases such as diabetic nephropathy, chronic kidney disease, and acute kidney disease (Wen et al., 2020). It consists of *Astragalus mongholicus* Bunge (3g), Panax notoginseng (1g), Angelica sinensis (3g), *Achyranthes bidentata* Blume (3g) and *Ecklonia kurome* Okamura (3g). The main component profile of A&P was analyzed by high performance liquid chromatography in our previous study (Hui et al., 2020). The formula was purchased from the affiliated traditional medicine hospital of Southwest Medical University, Luzhou City, Sichuan Province. The dosage for gavage is determined by referring to the conversion method of human and laboratory animals in “Pharmacological Experimental Methodology” edited by Professor Xu Shuyun. Mice daily dose = (human clinical daily dose × human defined body weight 70 kg × human and mouse body surface area conversion coefficient 0.0026) ÷ mouse defined body weight 20g. Therefore, the daily gavage dose of mice calculated by the formula is 1972 mg/kg/d. Bifidobacterium Lactobacillus triple live bacteria tablets (golden Bifidobacteria), its main components are Bifidobacterium, Lactobacillus bulgaricus and *Streptococcus thermophilus*. Bifidobacterium was purchased from Shenzhen Xinwanze Pharmaceutical Co., Ltd. The dose for gavage is also determined by referring to the conversion method of human and laboratory animals, and the daily gavage dose of Bifidobacterium in mice calculated by the formula is 303 mg/kg/d. Dissolve the powder of A&P and BB in distilled water, followed by intragastric administration of these drugs (200 μl) to mouse once a day. Mice in sham group were treated with gavage of equal volume of saline.

### Preparation of Drug-Containing Serum

A&P (1972 mg/kg/d), Bifidobacterium (303 mg/kg/d), and their combination were administered to male C57BL/6 mice (8 weeks of age, 20–24g body weight) by gavage for seven consecutive days. On the 7th day, blood was taken from the heart 2 h after the gavage, and stored at 4°C overnight. The next day, the blood was centrifuged at 3000 rpm for 10 min at 4°C to extract serum and collect all serum for subsequent cellular experiments. In the cell experiment, we used the prepared medicated serum instead of ordinary fetal bovine serum (10%).

### Simulate the Urotoxin Environment in RAW264.7 Cells

Add 200 ng/ml of Lipopolysaccharide (LPS) (L2630, Sigma-Aldrich, United States) to the complete medium of RAW264.7 cell to put the cells in an inflammatory stress state, then add indophenol sulfate (16926, Cayman, United States) 200 μM and *trans* aconitic acid (122750, Sigma, United States) 200 μM, respectively, to simulate the uremic environment.

### 5/6 Kidney Ligation Model (Nx5/6, CKD Model)

Male C57BL/6 mice (8 weeks of age and 22–25 g body weight) were classified into 5 groups: Sham, CKD, CKD + BB, CKD + A&P and CKD + BB + A&P group with 8 mice in each group. The CKD mouse model was constructed by directly ligating the upper and lower poles of left kidney after removal the right kidney 1 week later (Tan et al., 2019). Briefly, the right kidney was exposed and removed. One week later, the left kidney was exposed and the upper and lower poles were ligated with 3–0 non-absorbable suture. CKD mouse model can be obtained after six to 8 weeks of normal feeding after surgery. In this study, we determined that the model was successfully built at 7 weeks post-surgery, subsequently, the treatments were performed on these modeled mice for 4 weeks. Urine and feces of mice were collected by metabolic cage. All animal experiments were carried out according to the guidelines approved by the Animal Ethics Committee of Southwest Medical University.

### CCK-8 Analysis

Cells were seeded in 96-well plates at a density of 3000 per well, with 6 replicate wells per group. After 24 h of starvation culture, cells were treated with a drug-containing medium for 24 h, then the medium was aspirated, 100 μl of 10% CCK-8 reagent was added to each well, and incubated in an incubator for 3 h. The absorbance value of each well was measured at 450 nm by BioTeK reader, and the cell survival rate was calculated according to the results.

### Real-Time PCR

According to the instructions of the kit: Eastep qPCR Master Mix kit (LS2068, Promega, United States) is used to detect the mRNA expression levels of cells and kidneys; TRIzol Reagent (11596026, Invitrogen, United States) was used to extract total RNA from RAW264.7 cells and kidneys; RevertAid first-strand cDNA synthesis kit (K1622, Thermo Scientific, MA, United States) was used to reverse transcribe total RNA to cDNA; RT-PCR was performed with the Mastercycler ep Realplex2 real-time PCR system (LightCycle480II, Roche, United States). The relative expression of a given gene is calculated by the threshold cycle (CT) method. Primer sequences are listed in Table 1.

**TABLE 1:** List of primers used for Real-time PCR.

Gene	Species	Forward sequence (5′-3′)
		Reverse sequence (5′-3′)
Mincle	mouse	F: ACCAAATCGCCTGCATCC
		R: CACTTGGGAGTTTTTGAAGCATC
IL-1β	mouse	F: TTCAGGCAGGCAGTATCACTC
		R: GAAGGTCCACGGGAAAGACAC
IL-6	mouse	F: CTGCAAGAGACTTCCATCCAG
		R: AGTGGTATAGACAGGTCTGTTGG
TNF-α	mouse	F: CATCTTCTCAAAATTCGAGTGACAA
		R: TGGGAGTAGACAAGGTACAACCC
iNOS	mouse	F: CAGCTGGGTCGTACAAAC
		R: CATTGGAAGTGAAGCGTTT
MCP-1	mouse	F: CTGAGTTGACTCCTACTGTGGA
		R: TCTTCCCAGGGTCGATAAAGT
GAPDH	mouse	F: ACAGCAACAGGGTGGTGGAC
		R: TTTGAGGGTGCAGCGAACTT

### Enzyme-Linked Immunosorbent Assay

Collect CKD mouse serum and RAW264.7 cell culture supernatant. The concentration of IL-1β (Neobioscience, EMC001b), IL-6 (Neobioscience, EMC004), and TNF-α (Neobioscience, 500850) in serum and culture medium were detected according to the instructions of the ELISA kit. The absorbance value of each well was measured at 450 nm by BioTeK reader.

### Immunofluorescence Staining

After placing the frozen sections of the tissues at room temperature for 20 min, wash these sections for three times with PBS. Subsequently, block the samples with 10% goat serum for 30 min at 37°C, and then incubate the samples with rat anti-F4/80 primary antibody (Santa Cruz, 1:50, sc-52664) and mouse anti-Mincle primary antibody (Santa Cruz, 1:50, sc-390806) overnight at 4°C. The next day, wash these samples with PBS three times and conjugate with AlexaFluor488 conjugated anti-rabbit secondary antibody (CST, 1: 200, 4412S) and AlexaFluor647 conjugated anti-mouse secondary antibody (CST, 1: 200, 4410S) at room temperature for 1 h, followed by washing three times with PBS, then add diluted DAPI dropwise to stain the nuclei for 10 min in the dark. Finally, pictures were obtained with a confocal microscope (Nikon, Japan).

### Western Blotting

The total protein was extracted from cell and tissues using RIPA lysis buffer containing protease inhibitors and phosphatase inhibitors. Proteins (25 μg of each sample) were separated on a 10% SDS-PAGE gel and transferred to a PVDF membrane by wet blotting. After blocking in 5% milk, the membranes were incubated with the antibodies against p-p65 (Rabbit anti-mouse, sc-136548, 1:1000, Santa Cruz), p65 (Mouse anti-mouse, #6956, 1:1000, CST), Mincle (Mouse anti-mouse, sc-390806, 1:500, Santa Cruz), iNOS (Rabbit anti-mouse, 13120S, 1:1000, CST), ZO-1 (Rabbit anti-mouse, 61–7300, 1:1000, Life technologies), Occludin (Mouse anti-mouse, 33–1500, 1:1000, Life technologies), Claudin-1 (Rabbit anti-mouse, 71–7800, 1:1000, Life technologies) and GAPDH (Mouse anti-mouse, AB2000, 1:5000, Abiways) and β-actin (Rabbit anti-mouse, 1:50000, abcam) at 4°C overnight. The next day, membranes were subsequently incubated with the secondary antibody of peroxidase-conjugated goat anti-mouse IgG (ZB-2305, 1:3000) and goat anti-rabbit IgG (ZB-2301, 1:3000) for 1 h at room temperature. The band was analyzed by the gel imaging system. Gray intensity of the band was calculated by ImageJ software.

### Flow Cytometry Analysis

Fresh kidney tissue was digested with collagenase IV (RAW264.7 cells were digested with 0.25% trypsin-EDTA into cell suspension), then fixed at room temperature in 4% paraformaldehyde for 1 h. After washing with PBS for 2 times, cells were incubated with iNOS primary antibody (13120S, 1: 200, CST), CD206 primary antibody (ab64693, 1: 200, abcam, directly coupled antibody) and Mincle primary antibody (sc-390806, 1:50, Santa Cruz) at 4°C overnight. The next day, the cells were incubated with fluorescent secondary antibody for 1 h. After washing with PBS for 2 times, the cells were analyzed using a flow cytometer (FACSAria, BD Biosciences, United States), and the data were processed using FlowJo software (X 10.0.7).

### Detection of Renal Function

The urine and the tail blood of the mice were collected to detect the serum creatinine (C011-1-1, Nanjing Jiancheng Bioengineering Institute), urea nitrogen (C013-2-1, Nanjing Jiancheng Bioengineering Institute) and urine protein (C035-1-1, Nanjing Jiancheng Bioengineering Institute) following the instructions of corresponding kits.

### 16s DNA Detection of Microorganisms

The feces from each group were collected and stored in a special feces storage reagent every week. All of the samples were sent to Shanghai Ouyi Biomedical Technology Co., Ltd., for 16s DNA detection.

### Hematoxylin-Eosin Staining

The pre-treated tissue paraffin sections were stained in hematoxylin aqueous solution for 10 min, rinsed with running water for 5 min, then dehydrated in 100% alcohol, stained with Eosin (H&E, Beyotime, China) solution for 2–3 min, and finally transparent, and mounted. The tubulointerstitial injury index was graded as described previously (Radford et al., 1997). The degree of injury to the intestinal tissues was evaluated according to previous description (Chiu et al., 1970). Pictures were captured by the optical microscope (Eclipse 80i, Nikon, Japan).

### Sirius Red Staining

The paraffin slices were stained with the Sirius red stain at 37°C for one and a half hour, rinsed with running water for 10 min, then stained with hematoxylin aqueous solution for 10 min, followed by rinsing with running water for 10 min, finally dehydrated, transparent, and mounted. Images were captured by the optical microscope (Eclipse 80i, Nikon, Japan).

### Overexpression of Mincle by Plasmid Transfection

Extracting pcDNA3.1-Mincle and pcDNA3.1-vector plasmids from two Escherichia coli (purchased from GeneChem company) by using plasmid extraction kit (NucleoBond Xtra MiDi EF, 740420.50). Then, the plasmid and DNA transfection reagent (ZETA life) were mixed with complete medium and added to RAW264.7 cells for 48 h.

### Statistical Analysis

This study used SPSS22.0 and Prism 6 software for analysis. Data were presented as mean ± standard error. One-way analysis of variance (One-Way ANOVA) was used for multigroup comparison, followed by the Student-Newman-Keuls post-test. *P* < 0.05 was considered statistically significant.

## Results

### A&P Combined With Bifidobacterium Treatment Improves Kidney Function and Pathology in CKD Mice

**FIGURE 1:**
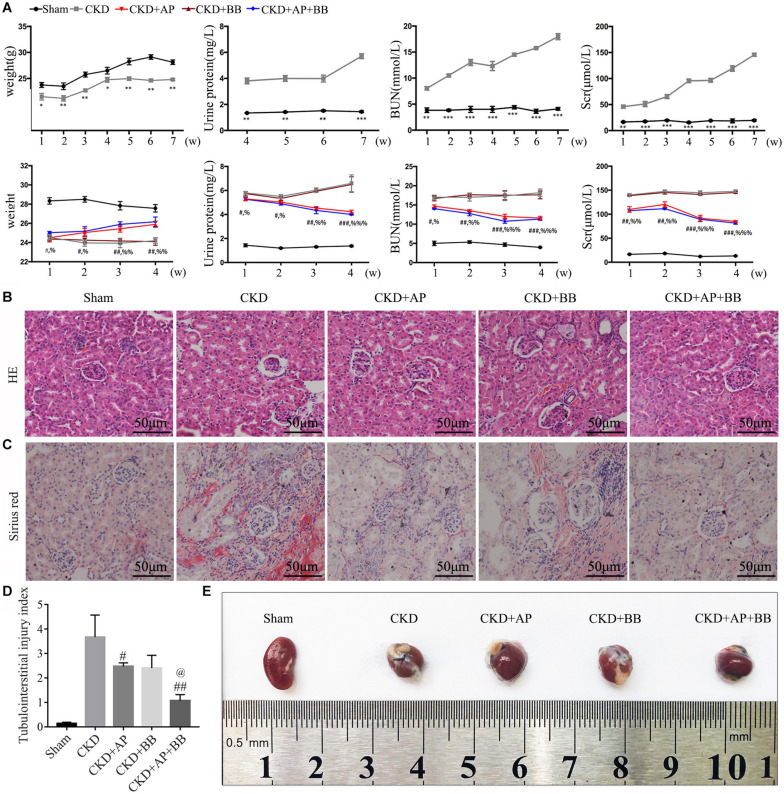
A&P combined with Bifidobacterium treatment improves kidney function and pathology in CKD mice. **(A)** The first row of Figure 1A is the indexes of body weight, urine protein, serum creatinine and urea nitrogen of the sham group and model group during 7 weeks of CKD modeling. The second row is the indexes of body weight, urine protein, serum creatinine and urea nitrogen of each group during 4 weeks of drug treatment; **(B)** Pathological H&E staining of kidney tissue in each group; **(C)** The Sirius staining of kidney tissue in each group. **(D)** The tubulointerstitial injury index; **(E)** The kidneys from each group of mice; ^∗^*P* < 0.05, ^∗∗^*P* < 0.01, ^∗∗∗^*P* < 0.001, vs. Sham group; ^#^*P* < 0.05, ^##^*P* < 0.01, ^###^*P* < 0.001, vs. CKD group; ^%^*P* < 0.05, ^%%^*P* < 0.01, ^%%%^*P* < 0.001, vs. CKD group; ^@^*P* < 0.05, vs. CKD + AP group.

During the 7 weeks of modeling, compared with the sham group, the blood creatinine, urea nitrogen and urine protein in the 5/6 kidney ligation model group were all increased, and the body weight was reduced. In contrast, the blood creatinine, urea nitrogen and urine protein in A&P + Bifidobacterium treated mice were significantly decreased, and their body weight were increased. However, mice using Bifidobacteria alone did not exhibit significant improvement on renal function (Figure 1A). H&E and Sirius red stainings were employed to detect the protective effect of A&P on kidney of CKD model. The H&E staining results showed that compared with the sham group, the kidney in CKD model mice present glomerular atrophy and deformation, renal tubular expansion as well as necrosis and shedding of renal tubular epithelial cells. After treatment with A&P + Bifidobacteria, the expansion of renal tubules in CKD mice was significantly reduced, and the glomerular morphology was restored (Figure 1B). The result of Sirius red staining demonstrated that, compared with the sham group, the positive area of sirius red staining in kidney of CKD mice was increased, indicating severe renal fibrosis, accompanied by glomerular atrophy and renal tubular expansion. However, compared with CKD group, kidney in A&P and Bifidobacterium combination treatment group showed significantly reduction of fibrosis and other pathological damages (Figure 1C). The tubulointerstitial injury index was graded as described previously (Figure 1D). Figure 1E shows the morphology of each group of kidneys. These results suggested that A&P combined with bifidobacterium treatment can significantly improve the body weight and renal function in CKD mice.

### A&P Combined With Bifidobacterium Treatment Improves Renal and Systemic Inflammation in CKD Mice

**FIGURE 2:**
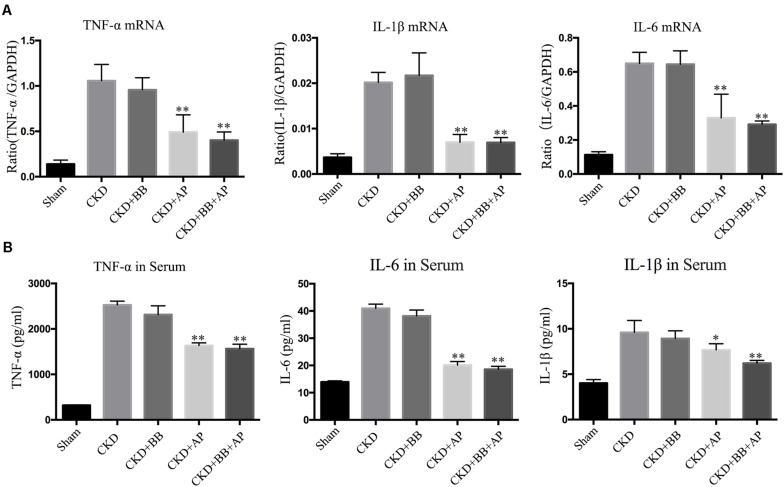
A&P combined with bifidobacterium treatment improves renal and systemic inflammation in CKD mice. **(A)** Real-time PCR detected the mRNA expression of inflammatory indicators (IL-1β, IL-6, TNF-α) in kidney of each group; **(B)** ELISA detected the secretion of inflammatory factors in serum of each group. ^∗^*P* < 0.05, ^∗∗^*P* < 0.01, vs. CKD group.

To investigate the effects of A&P + Bifidobacterium on inflammation in CKD mice, we performed Real-time PCR and ELISA to exam the expression and secretion of inflammatory cytokines. The PCR results showed that, compared with CKD group, A&P and Bifidobacterium combination significantly reduced mRNA expression of inflammatory indicators (IL-1β, IL-6, and TNF-α) in kidney of CKD mice (Figure 2A), whose inhibitory effect was better than using Bifidobacterium alone. Moreover, the ELISA results demonstrated that, compared with CKD group, A&P and Bifidobacterium combination strongly decreased secretion of inflammation indicators (IL-1β, IL-6, and TNF-α) in serum of CKD mice (Figure 2B). These results suggested that the combination of A&P and Bifidobacterium can effectively improve the renal and systemic inflammation in CKD mice.

## A&P Combined With Bifidobacterium Treatment Restored the Intestinal Barrier of CKD Mice

**FIGURE 3:**
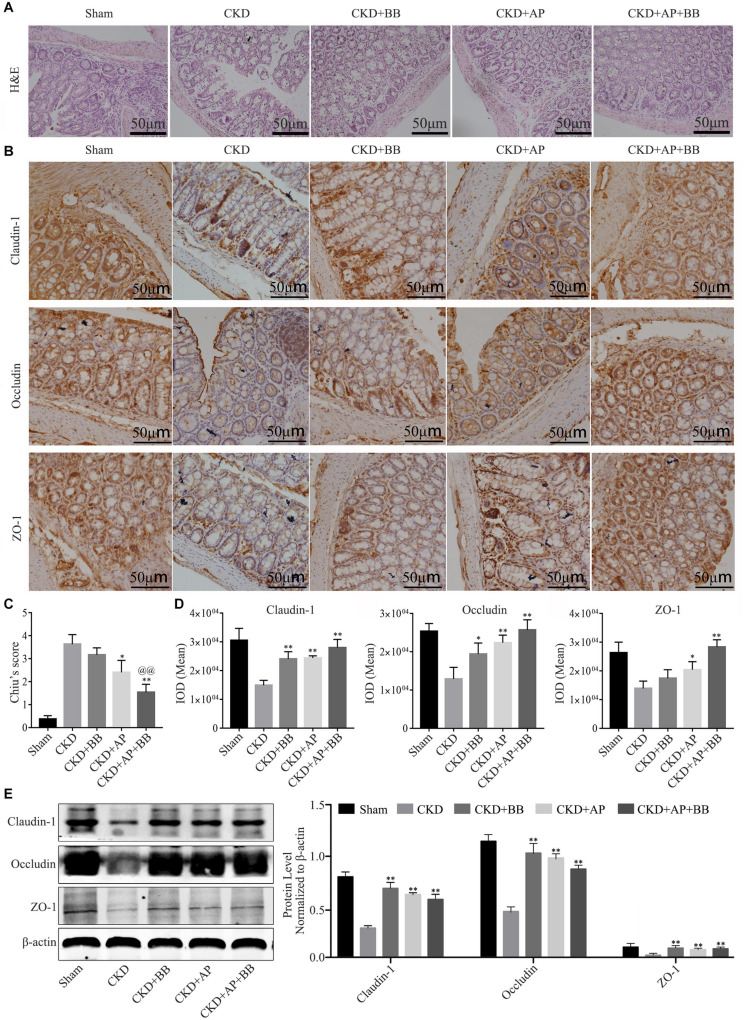
A&P combined with Bifidobacterium treatment can restore the intestinal barrier of CKD mice. **(A)** H&E staining of intestine in each group (× 200); **(B)** Immunohistochemical staining of Claudin-1, Occludin and ZO-1 in intestines of each group (× 200); **(C)** Intestinal damage was evaluated in each group according to Chiu’s score; **(D)** The mean IOD of Claudin-1, Occludin-1 and ZO-1 IHC in **(B)**; **(E)** Western blot detected the protein levels of Claudin-1, Occludin and ZO-1 in intestine of each group. ^∗^*P* < 0.05, ^∗∗^*P* < 0.01, vs. CKD group; ^@@^*P* < 0.01, vs. CKD + AP group.

Intestinal barrier is essential for maintaining intestinal function, we therefore detected the expression of indicators of intestinal barrier in intestines of each group. The H&E staining results showed that, compared with the CKD group, A&P and Bifidobacterium combination reduced the intestinal damage of the CKD mice (Figure 3A). Immunohistochemical staining results indicated that the ZO-1, Occludin-1 and claudin-1 proteins in intestine of Sham group were evenly distributed on the top of intestinal epithelial cells with a honeycomb or dot shape. However, these proteins were significantly reduced in intestine of CKD group. Compared with CKD group, the protein levels of ZO-1, Occludin-1 and claudin-1 in intestine of A&P and Bifidobacterium combination group were strongly recovered (Figure 3B). Intestinal damage was evaluated in each group according to Chiu’s score (Figure 3C). The mean IOD of Claudin-1, Occludin-1 and ZO-1 IHC were illustrated in Figure 3D. The western blot results also proved the up-regulation of ZO-1, Occludin-1 and claudin-1 protein levels in A&P and Bifidobacterium combination group compared with the CKD model group (Figure 3E). These results suggested that A&P combined with Bifidobacterium had a protective effect on intestinal barrier of CKD mice.

### A&P Combined With Bifidobacterium Treatment Has a Positive Effect on the Intestinal Flora of CKD Mice

**FIGURE 4:**
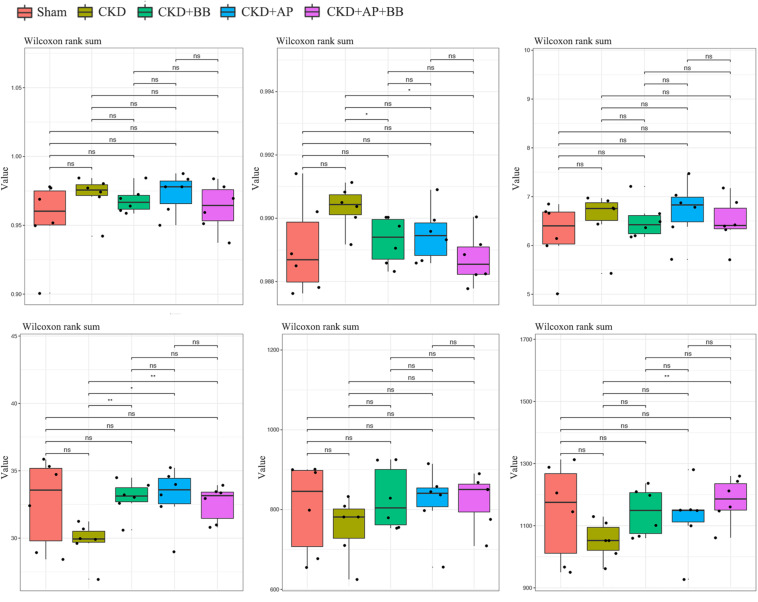
A&P combined with Bifidobacterium treatment has a positive effect on the intestinal flora of CKD mice. 16s alpha diversity related boxplot analysis of mouse feces from each group. ^∗^*P* < 0.05, ^∗∗^*P* < 0.01.

To test whether A&P combined with Bifidobacterium improved the intestinal flora of CKD mice, we collected mouse feces and subjected to microbiological 16s DNA detection. Among them, alpha diversity is related to boxplot analysis. This method calculates the diversity index of the samples between each group and within the group on the basis of a uniform data depth, and draws a boxplot diagram of the diversity index of each group. Microbial 16s DNA test results showed that compared with CKD group, A&P combined with Bifidobacteria treatment recovered the intestinal flora to normal in CKD mice, which was better than using A&P or Bifidobacteria alone (Figure 4). These results showed that A&P combined with Bifidobacterium treatment has a positive effect on intestinal flora of CKD mice than treating with A&P or Bifidobacterium alone.

### A&P Combined With Bifidobacterium Inhibited the Mincle/NF-κB Signaling Pathway and M1 Macrophage Activity in the Kidney of CKD Mice

**FIGURE 5:**
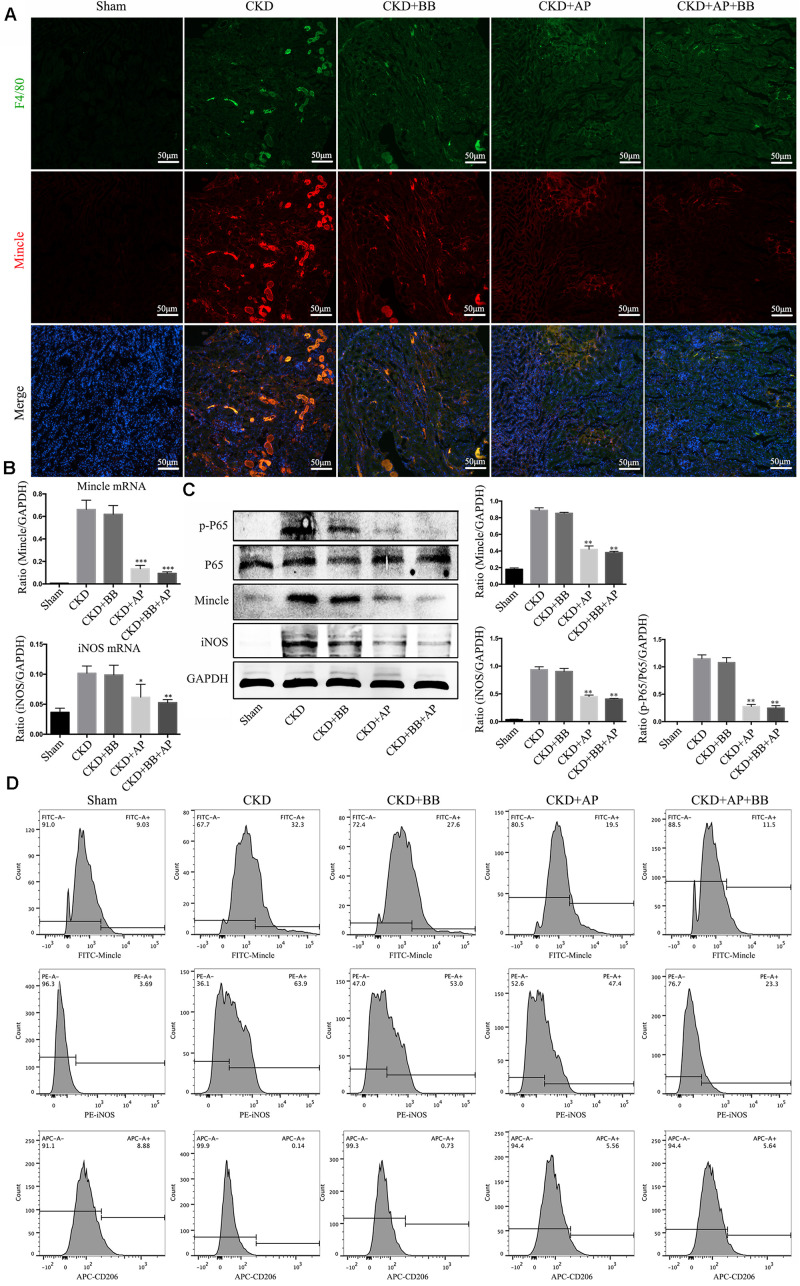
A&P combined with Bifidobacterium inhibited the Mincle/NF-κB signaling pathway and M1 macrophage activity in kidney of CKD mice. **(A)** The immunofluorescence staining detected the expression and location of F4/80 (Green) and Mincle (Red) in kidney of each group (× 200); **(B)** The mRNA expression of Mincle and iNOS in kidney of each group detecting by Real-time PCR; **(C)** Western blot detected the protein levels of Mincle/iNOS/p-P65/p65 in kidney of each group; **(D)** Flow cytometry was performed to test the levels of Mincle/iNOS/CD206 in kidney of each group. ^∗^*P* < 0.05, ^∗∗^*P* < 0.01, ^∗∗∗^*P* < 0.001, vs. CKD group.

To investigate the underlying mechanism of A&P and Bifidobacterium combination in protection of kidney in CKD, we detected the Mincle/NF-κB signaling pathway in the kidney of each group. The immunofluorescence results showed that the macrophage (marker F4/80: green spots) largely infiltrated in the CKD kidney, and Mincle (red spots) was also strongly increased in the CKD kidney. Interestingly, the green and red spots were largely merged together, which means that Mincle is mainly expressed on macrophage. Compared with CKD group, the macrophage infiltration in the kidney of A&P + Bifidobacterium treated mice was significantly reduced, and the expression of Mincle was also down-regulated (Figure 5A). Moreover, Real-time PCR results showed that A&P and Bifidobacterium combination effectively inhibited the mRNA expression of Mincle and iNOS (Figure 5B). Western blot results also demonstrated that the protein levels of Mincle and iNOS as well as the phosphorylated NF-κB were significantly reduced in A&P and Bifidobacterium treatment group, which was consistent with the results of immunofluorescence (Figure 5C), but levels of these protein in mice treated with Bifidobacterium alone did not changed significantly. Subsequently, the flow cytometry was employed to exam the changes of macrophage polarization in each group, and the results showed that the expression of Mincle and M1 macrophage markers (iNOS) in kidney of CKD mice were increased, while A&P combined with Bifidobacterium treatment significantly decreased Mincle and iNOS. On the other hand, the expression of M2 macrophage surface marker (CD206) in kidney of CKD mice was decreased, which was recovered by A&P and Bifidobacterium combination (Figure 5D). These above results suggested that A&P and Bifidobacterium combination can strongly inhibit the activity of M1 macrophages and increase the activity of M2 macrophages by suppressing the Mincle/NF-κB pathway.

### A&P Combined With Bifidobacterium Inhibited the Mincle/NF-κB Signaling Pathway in the Intestine of CKD Mice

**FIGURE 6:**
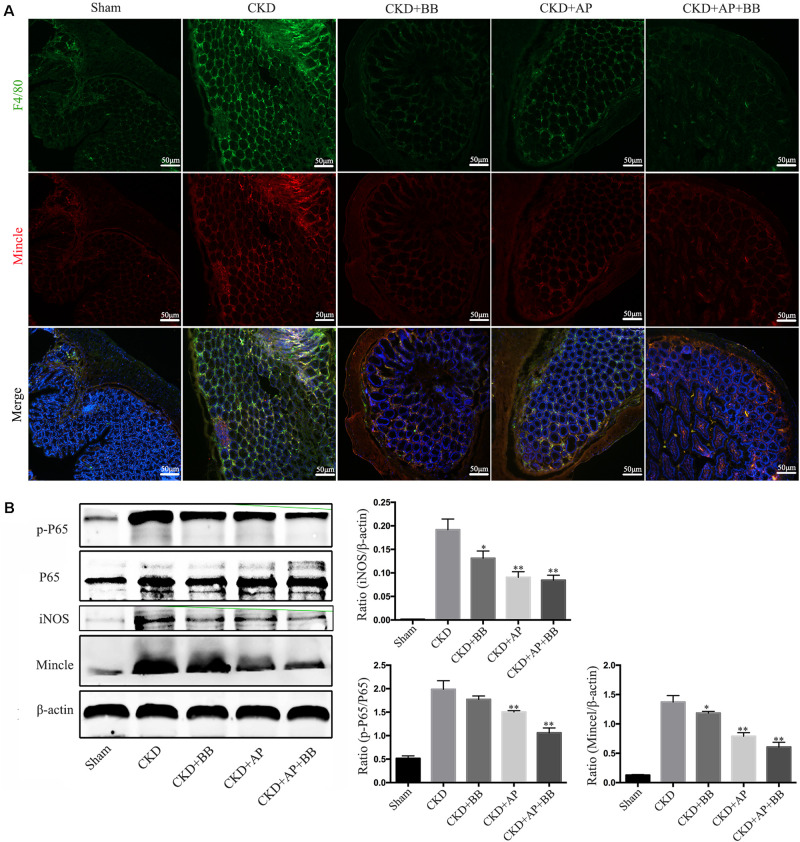
A&P combined with Bifidobacterium inhibited the Mincle/NF-κB signaling pathway in the intestine of CKD mice. **(A)** The immunofluorescence staining detected the expression and location of F4/80 (Green) and Mincle (Red) in intestine of each group (× 200); **(B)** Western blot detected the protein levels of Mincle/iNOS/p-P65/p-65 in intestine of each group. ^∗^*P* < 0.05, ^∗∗^*P* < 0.01, vs. CKD group.

We further detected the Mincle/NF-κB signaling pathway in intestine of each group. The immunofluorescence results showed that the levels of F4/80 and Mincle were significantly increased in intestine of CKD mice, however, A&P and Bifidobacteria combination largely reduced macrophage infiltration and Mincle expression (Figure 6A). Western blot results showed that compared with the CKD group, the protein levels of Mincle, iNOS and phosphorylated NF-κB were significantly down-regulated in A&P and Bifidobacterium combined group, which is better than using A&P or Bifidobacterium alone (Figure 6B). These data suggested that A&P combined with Bifidobacterium can inhibit the Mincel/NF-κB signaling in intestine of CKD mice.

### A&P and Bifidobacterium Containing Serum Inhibited Inflammation in RAW264.7 Cells Induced by LPS and Indophenol Sulfate Induced

**FIGURE 7:**
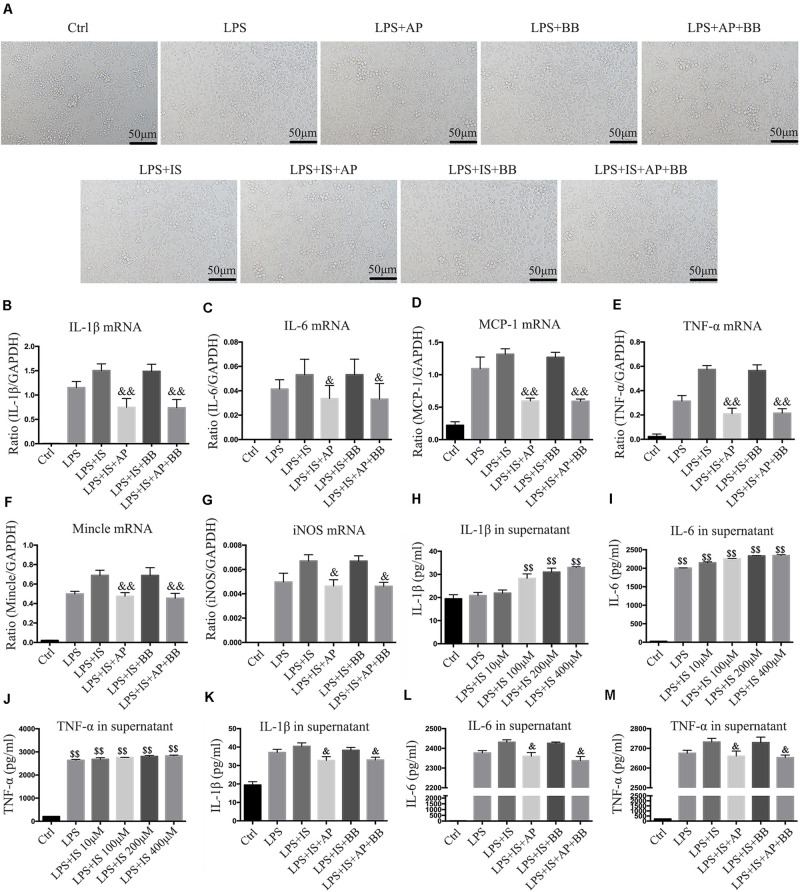
A&P and Bifidobacterium containing serum inhibited LPS and indophenol sulfate induced inflammation in RAW264.7 cells. **(A)** The cell morphology of each group was observed under an microscope (×200); **(B–G)**. Real-time PCR was performed to detect the mRNA expression of inflammatory factors (IL-1β, IL-6, TNF-α, MCP-1), Mincle and iNOS in RAW264.7 cells after modeling with LPS and indophenol sulfate; **(H–M)**. ELISA was used to detect the secretion of inflammatory factors (IL-1β, IL-6, TNF-α) in supernatant of RAW264.7 cells after modeling with LPS and indophenol sulfate. ^&^*P* < 0.05, ^&⁣&^*P* < 0.01, vs. LPS + IS Group; ^$$^*P* < 0.01, vs. Ctrl Group.

To simulate the uremic environment, we used LPS and indophenol sulfate to stimulate the RAW264.7 macrophage. Morphological observation revealed that normal RAW264.7 cells were smooth and round, with high density and distinct particles. Compared with the LPS group or LPS + IS group, A&P and Bifidobacterium containing serum significantly reduced protrusions and pseudopods on cells, and the overall cell morphology was improved (Figure 7A). Real-time PCR results showed that, compared with the LPS or LPS + IS groups, A&P and Bifidobacterium containing serum largely decreased the mRNA expression of inflammatory indicators (IL-1β, IL-6, TNF-α, MCP-1), Mincle and iNOS, however, the cells treated with Bifidobacterium containing serum alone did not inhibited the mRNA expression of these indicators (Figures 7B–G). ELISA results demonstrated that, compared with the LPS group, administration of LPS plus different concentration of IS significantly up-regulated the secretion of inflammatory indicators (IL-1β, IL-6, TNF-α) in supernatant (Figures 7H–J), and the IS concentration of 200 μM was used in the subsequent experiments. Then, we found that, compared with the LPS + IS group, A&P and Bifidobacterium containing serum strongly reduced the secretion of inflammatory indicators, but the cells treated with Bifidobacterium containing serum alone did not inhibited the secretion of these indicators (Figures 7K–M). These results suggested that A&P and bifidobacterium containing serum can improve the macrophage morphology and inflammation under indophenol sulfate induced uremic environment.

### A&P and Bifidobacterium Containing Serum Suppressed Inflammation in RAW264.7 Cells Induced by LPS and *Trans* Aconitic Acid Induced

**FIGURE 8:**
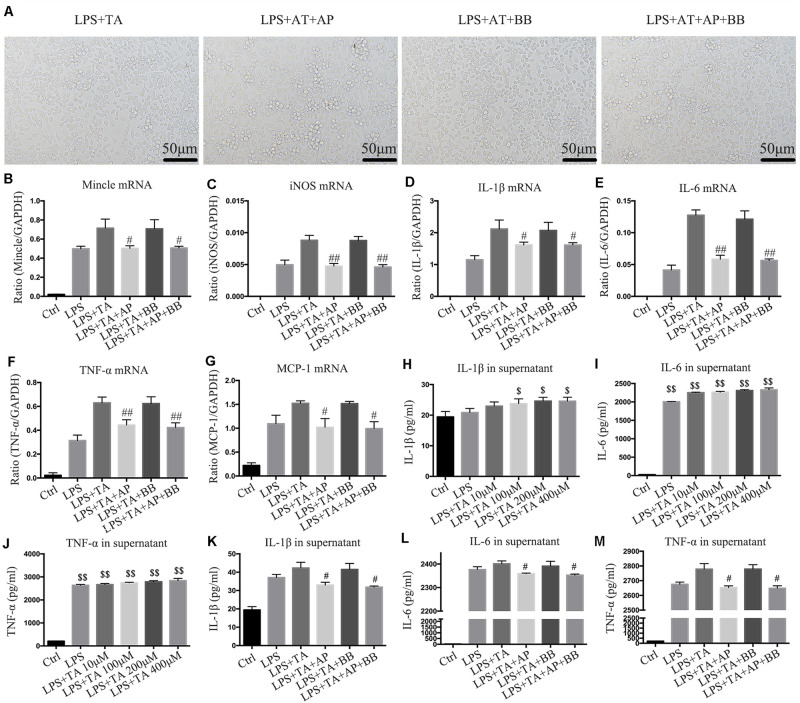
A&P and Bifidobacterium containing serum suppressed LPS and *trans* aconitic acid induced inflammation in RAW264.7 cells. **(A)** The cell morphology of each group was observed under an microscope (×200); **(B–G)** Real-time PCR was performed to detect the mRNA expression of inflammatory factors (IL-1β, IL-6, TNF-α, MCP-1), Mincle and iNOS in RAW264.7 cells after modeling with LPS and *trans* aconitic acid; **(H–M)** ELISA was used to detect the secretion of inflammatory factors (IL-1β, IL-6, TNF-α) in supernatant of RAW264.7 cells after modeling with LPS and *trans* aconitic acid. ^#^*P* < 0.05, ^##^*P* < 0.01, vs. LPS + IS Group; ^$^*P* < 0.05, ^$$^*P* < 0.01, vs. Ctrl Group.

We used another urotoxin (*trans* aconitic acid) to simulate uremic environment. We found that, compared with the LPS group or LPS + TA group, A&P and Bifidobacterium containing serum largely decreased protrusions and pseudopods on cells, and the overall cell morphology was improved (Figure 8A). Real-time PCR results demonstrated that, compared with the LPS or LPS + TA groups, A&P and Bifidobacterium containing serum significantly reduced the mRNA expression of inflammatory indicators, Mincle and iNOS, however, the cells treated with Bifidobacterium containing serum alone did not inhibited the mRNA expression of these indicators (Figures 8B–G). ELISA results determined the 200 μM concentration of TA was used in the subsequent experiments (Figures 8H–J). We then found that, compared with the LPS + TA group, A&P and Bifidobacterium containing serum strongly reduced the secretion of inflammatory indicators, but the cells treated with Bifidobacterium containing serum alone did not inhibited the secretion of these indicators (Figures 8K–M). These results also suggested that A&P and bifidobacterium containing serum can improve the macrophage morphology and inflammation under *trans* aconitic acid induced uremic environment.

### Overexpression of Mincle Abrogated A&P and Bifidobacterium Containing Serum Inhibited Inflammation in LPS + IS Stimulated RAW264.7 Cells

**FIGURE 9:**
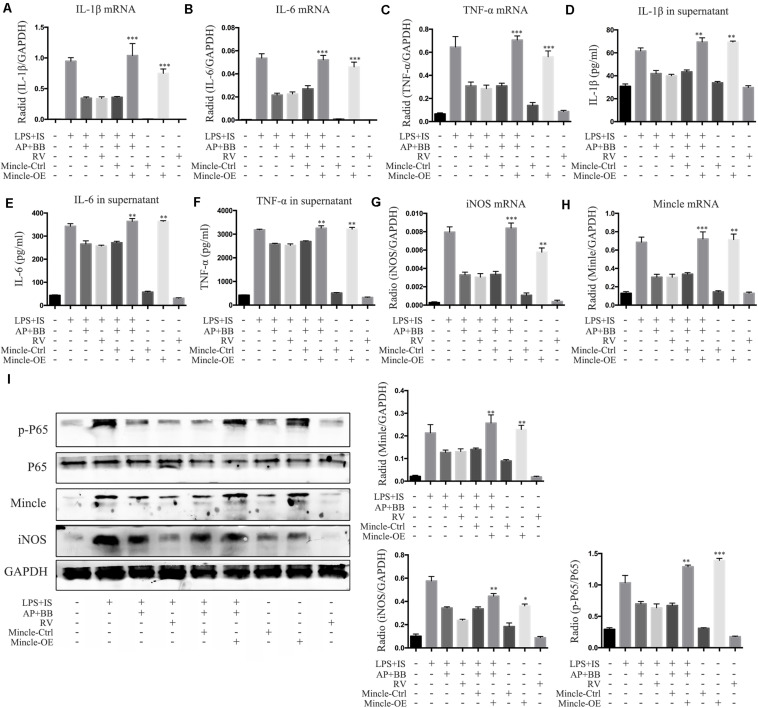
Overexpression of Mincle recovered A&P and Bifidobacterium containing serum inhibited inflammation in LPS + IS stimulated RAW264.7 cells. **(A–C)** Real-time PCR was performed to detect the mRNA expression of inflammatory indicators (IL-1β, IL-6, TNF-α) in RAW264.7 cells after overexpression of Mincle; **(D–F)** ELISA was used to detect the secretion of inflammatory factors (IL-1β, IL-6, TNF-α) in supernatant of RAW264.7 cells after overexpression of Mincle; **(G,H)** The mRNA expression of iNOS and Mincle in RAW264.7 cells; **(I)** The protein levels of Mincle/iNOS/p-P65/p-65 in RAW264.7 cells after overexpression of Mincle. ^∗^*P* < 0.05, ^∗∗^*P* < 0.01, ^∗∗∗^*P* < 0.001, vs. LPS + IS + A&P + BB Group.

To further explore the relationship between Mincle and inflammatory response in macrophage, we overexpressed Mincle in A&P combined Bifidobacterium treatment group by plasmid transfection, and set up a positive control group (resveratrol (RV) 30 μM). Real-time PCR and ELISA results showed that after overexpression of Mincle in treatment group, the expression and secretion of inflammatory cytokines were significantly increased (Figures 9A–H). Western blot results demonstrated that the protein level of Mincle in the plasmid-transfected group (Mincle-OE) was strongly increased, which activated the Mincle/NF-κB signaling and M1 macrophage in A&P and Bifidobacterium containing serum treated inflammatory RAW264.7 cells (Figure 9I). These results suggested that the anti-inflammatory effect of A&P and Bifidobacterium in macrophage is dependent on the inhibition of the Mincle signaling pathway.

## Discussion

Current treatments for chronic kidney disease mainly include using drugs to control proteinuria, blood sugar, blood pressure, calcium and phosphorus metabolism and adjust diet. When CKD progresses to the uremic stage of late renal failure, dialysis or kidney transplantation is the final choice. With the widespread clinical application of traditional Chinese medicine in the treatment of CKD (Zhong et al., 2013, 2015; Zhang et al., 2014), people have a new understanding of traditional Chinese medicine. Our team used the theory of “atrophic lung disease” from the traditional Chinese medicine book – “The Golden Chamber” to propose the theory of “flaccidity caused by the disorder of the kidney” in CKD. Based on the above-mentioned traditional Chinese medicine theory, our team created the A&P formula, containing *Astragalus mongholicus* Bunge, Panax notoginseng, Angelica sinensis, *Achyranthes bidentata* Blume and *Ecklonia kurome* Okamura. Although A&P can effectively treat CKD in clinic in Chinese, its pharmacological mechanism for the treatment of CKD is not clear.

With the development of intestinal microbial medicine, Meijers et al. proposed the concept of “intestinal-kidney axis” in 2011 (Meijers and Evenepoel, 2011), which initially clarified the relationship between chronic kidney disease and intestinal microecology. Ritze et al. also proposed the concept of “intestinal-renal syndrome,” revealing the vicious cycle between the occurrence and development of CKD and the increase of intestinal permeability (Ritz, 2011). Over the past few decades, clinical understanding of the structure and function of the gut microbiota has greatly enriched with the advancement of culture-related technologies (Hooper and Macpherson, 2010). The intestinal flora can maintain a very dynamic symbiosis of bacteria and interact with other systems of the human body to plays an important role in nutrition and energy metabolism, immune regulation and host defense (Round and Mazmanian, 2009; Morgan et al., 2012; Qin et al., 2012; Kamada et al., 2013; Ridaura et al., 2013). The harmful effects of intestinal flora in critical illness are multifaceted and can be divided into three areas: destruction of the microbial barrier, loss of colonization resistance and metabolic disorders (Ubeda et al., 2010; Andersen et al., 2017). Intestinal microflora dysregulation may lead to bacterial translocation by increasing intestinal permeability and inducing mucosal immune dysfunction (Anders et al., 2013). When the damage of bacteria’s permeability to the intestinal mucosa increases to a certain degree, it activate the intestinal mononuclear macrophages and promote cells to release a large number of toxic cytokines and chemicals such as inflammatory factors and oxygen free radicals, which aggravate the damage to the protective barrier of the intestinal mucosa (Strzępa and Szczepanik, 2013). This repeated cycle leads to chronic inflammation of the system. The persistent chronic inflammation is one of the most important independent factors that predict the poor prognosis of CKD patients.

Recently, the interaction between intestinal microflora and kidneys has received increasing attention. Dietary supplement of probiotics was widely used to improve intestinal microenvironment and reduced production of urotoxin (Wu et al., 2016; Tao et al., 2019). Probiotic is the ingredients of indigestible food that can beneficially affect the host by increasing the number of specific bacteria and changing the composition of the microbiome (Gibson and Roberfroid, 1995; Nakabayashi et al., 2011; Iwashita et al., 2018). It also has been reported that probiotic can improve the kidney function of humans and animals by improving the intestinal microenvironment (Ranganathan et al., 2010; Furuse et al., 2014; Yoshifuji et al., 2016), but using the probiotic alone to treat CKD is highly controversial, therefore, in this study we try to explore the effects of treatment with combination of probiotic and traditional Chinese medicine on CKD, and the underlying mechanism.

In this study, the data demonstrated that A&P combined with Bifidobacterium can significantly reduce the urine protein, serum creatinine and urea nitrogen, and increase the body weight in 5/6 nephrectomy induced CKD mice. In addition, we found that A&P combined with Bifidobacterium can also effectively improve the intestinal flora of CKD mice by using 16s DNA detection of microorganisms. It is worth noting that A&P combined with Bifidobacterium significantly inhibited the inflammation in kidney and intestine of CKD mice and also down-regulated the concentration of inflammatory factors in blood. All the above results showed that the treatment effects of A&P combined with Bifidobacteria on CKD is better than that of Bifidobacteria treatment alone. However, although the effects of combination of A&P and BB is not significantly different from A&P treatment alone on some indicators, the combination of A&P and BB is better than A&P treatment alone in the damage scores of the kidney and intestine, as well as the intestinal inflammation, it may be because of BB is a probiotic, which mainly improves the intestinal microecology. On the other hand, the effect of combination of A&P and BB on certain indicators is not statistically different compared with A&P alone, which may be related to the strong inhibitory effect of A&P, but in general, the combination of A&P and BB has a better effect on improving the kidney and intestinal damage induced by CKD. Taken together, these findings proved that A&P combined with Bifidobacterium can strongly improve renal function, reduce renal and intestinal inflammation as well as improve intestinal flora, suggesting that A&P combined with Bifidobacterium protects kidney in CKD may partially through regulating intestinal microenvironment.

Macrophages are important innate immune cells with phagocytic functions. In the past few decades, people have gradually discovered that macrophages have a high degree of diversity (Yonit et al., 2015; Wynn and Vannella, 2016). It plays a complex role in various physiological and pathological processes, such as tissue development and homeostasis, host defense, tissue damage and repair, and regulation of fibrosis. Recent studies have shown that macrophage plays an important role in the evolution of AKI to CKD (Black et al., 2018). But the research on the relationship between dynamic changes of macrophage and the development of CKD is not clear. Therefore, this study intends to study the changes in the polarization of macrophage during the development of renal and intestinal injury in CKD through *in vivo* and *in vitro* experiments (Honarpisheh et al., 2018; Komada et al., 2018). Mincle is a transmembrane recognition receptor on innate immune cells, which is expressed in macrophages and critical to maintain the M1 phenotype of macrophages and triggering M1 macrophage-mediated kidney inflammation (Lv et al., 2017). Our results confirmed that the combination of A&P and Bifidobacterium can reduce the activity of M1 macrophages and increase the activity of M2 macrophages by inhibiting Mincle in kidney of CKD. Studies have shown that drugs can block NF-κB signaling in intestinal epithelial cells and macrophages, and improve acute and chronic murine colitis model (Yonit et al., 2015; Lee et al., 2020). Our results also proved that A&P combined with Bifidobacterium can improve the intestinal inflammatory state by adjusting the polarization of macrophage. As we know, NF-κB is an important transcription factor that regulates the expression of pro-inflammatory cytokines, and Mincle is strictly regulated by the TLR4/NF-κB signaling (Zhao et al., 2014; Tan et al., 2020). The results of this study showed that combination of A&P and Bifidobacterium can significantly reduce the activation of NF-κB through targeted modulation of Mincle to improve the inflammation in kidney and intestine.

## Conclusion

In summary, this study indicated that A&P combined with Bifidobacterium can significantly improve the renal function, down-regulate renal and intestinal inflammation, improve intestinal microenvironment in CKD, which may through inhibiting the Mincle/NF-κB signaling and suppressing inflammatory response in macrophage. This study provides a new insight in treatment of CKD with traditional Chinese medicine, and may optimize the application of A&P in clinical treatment of CKD.

## Data Availability Statement

The original contributions presented in the study are included in the article/supplementary material, further inquiries can be directed to the corresponding author.

## Ethics Statement

The animal study was reviewed and approved by the Ethics Committee of Southwest Medical University.

## Author Contributions

WL, TR-Z, FJ-M, and DH conceived and designed the experiments. TR-Z, DH, LJ-C, WD, and ZX performed the experiments. TR-Z, WX-J, and DH wrote the manuscript. All authors contributed to the article and approved the submitted version.

## Conflict of Interest

The authors declare that the research was conducted in the absence of any commercial or financial relationships that could be construed as a potential conflict of interest.

## Supplementary Material

Supplementary Figure 1 Cell viability test of indophenol sulfate and *trans* aconitic acid on RAW264.7 cells.
