# Supplementary figures and images for: Astragalus mongholicus Bunge and Panax Notoginseng Formula (A&P) Combined With Bifidobacterium Contribute a Renoprotective Effect in Chronic Kidney Disease Through Inhibiting Macrophage Inflammatory Response in Kidney and Intestine
Source: Front Physiol. 2020 Nov 27;11:583668. doi: 10.3389/fphys.2020.583668 (PMC7729014; doi:10.3389/fphys.2020.583668)

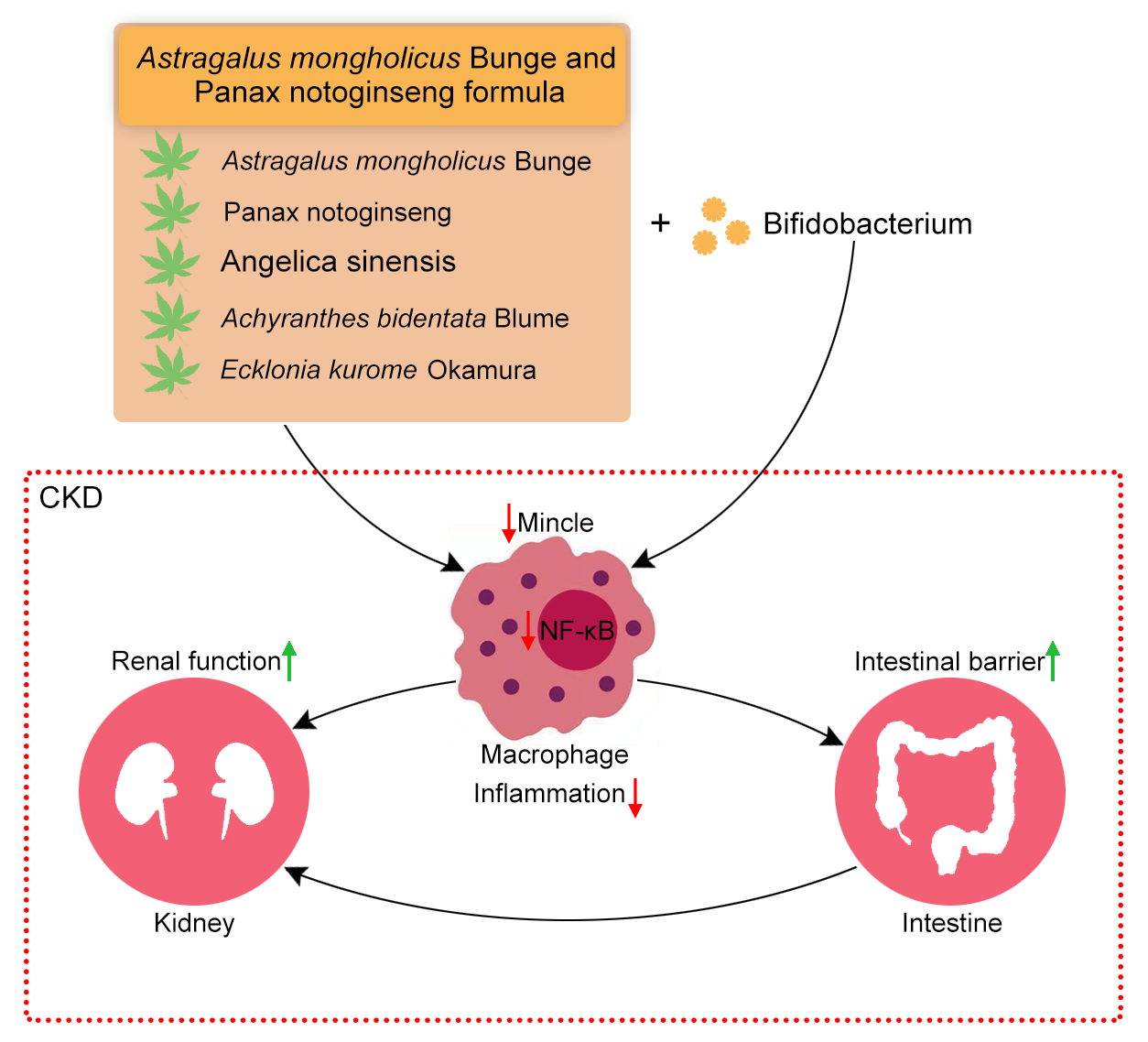

Supplement: Supplementary file 1 [file Image_2.TIF]

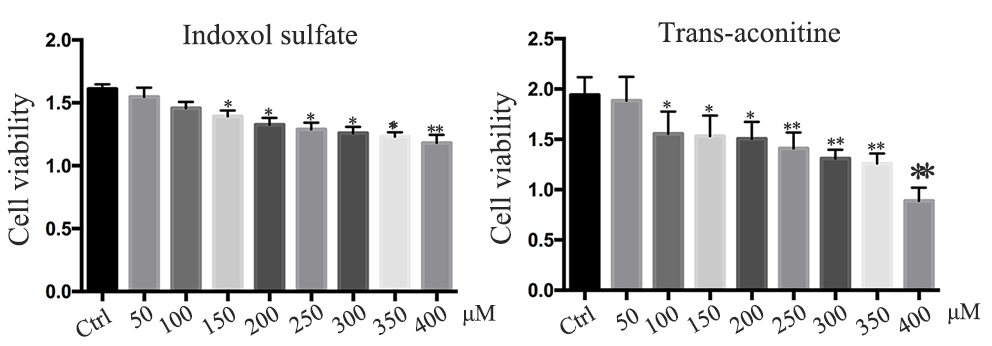

Supplement: Supplementary Figure 1 — Cell viability test of indophenol sulfate and trans aconitic acid on RAW264.7 cells. [file Image_1.TIF]
